# A Tribute to Dr. Willy Lens

**DOI:** 10.5334/pb.355

**Published:** 2016-07-13

**Authors:** Maarten Vansteenkiste, Lennia Fernandez, Athanasios Mouratidis

**Affiliations:** 1 Ghent University, BE; 2 Department of Psychology, Pontifical University Catholic Peru, PE; 3 Department of Psychology, Hacettepe University, TR

## Abstract

Dr. Willy Lens, born on December 10^th^, 1943, passed away on August 29^th^, 2014. With his passing, the motivation community has lost a seminal member, a mentor, and a friend. Dr. Lens – a Fellow of the Association for Psychological Science and Founding Fellow of the American Educational Research Association – made fundamental contributions to the study of motivation both through his own work and through his caring and thoughtful mentorship of a large community of scholars. With this tribute, we want to honor Dr. Willy Lens’ significance to psychology and education as well as his positive influence, both personally and professionally, on the lives of dozens of scholars. With his contagious enthusiasm and caring mentorship, Willy was an example for our academic community and with this tribute we express our gratitude for the privilege to have collaborated with him.

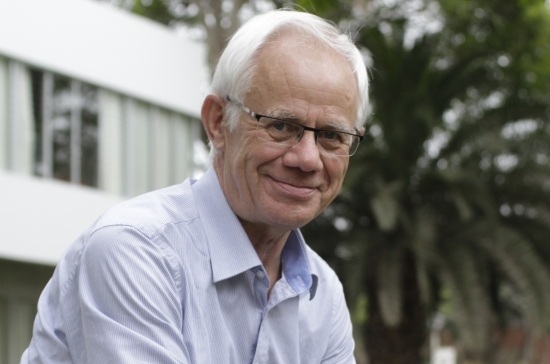



Dr. Willy Lens passed away on August 29^th^, 2014 while vacationing with his wife and family. His death was abrupt and has deeply touched the family, friends, and scholars who had the privilege to have known Willy. In this tribute, we do not only want to look back at his academic career, interests, and accomplishments but we also want to highlight some of the remarkable features of Willy’s personality. In the weeks after Willy’s death, dozens of friends and colleagues, both in Belgium and worldwide, wrote personal memories that were brought together in a condolence booklet that was given to his family. This tribute is meant to reflect those personal memories and souvenirs of the many friends and colleagues whose lives and careers were deeply influenced by Willy.

## Dr. Willy Lens’ Academic Career

Willy began his academic career in Leuven authoring a chapter with his mentor J. R. Nuttin in a book edited by Paul Fraisse and Jean Piaget. He then continued his research on Time Perspective and Achievement Motivation as a post-doctoral student with John Atkinson at the University of Michigan, Ann Arbor during 1972–1973. After returning to Belgium as a Junior Professor, he quickly was promoted to Full Professor and Chair of the Center for Motivation and Time Perspective at KU Leuven. Over the years, the center gradually grew, being a place for lifelong learning for the many doctoral students he trained.

As a prolific scholar, Willy published over 120 articles in 11 languages. The list of his publications even grew after his death – with four articles published in 2014 and another seven in 2015. A few more papers authored by Willy are in the review process and will be published in the future with the aid of his collaborators. To illustrate, given Willy’s significant involvement in the dissertations of Pedro Cordeiro and Nicky Roman, he is involved as a co-author in their recent publications (Cordeiro, Paixão, Lens, Lacante & Luyckx, this issue; Roman, Davids, Moyo, Schilder, Lacante, & Lens, 2015). Although he worked internationally throughout his academic career, he always remained connected to his home country and his university, the KU Leuven (Belgium). He published a significant amount of work in Dutch, including two Dutch textbooks; one specifically in the study of motivation in collaboration with Eric Depreeuw (Lens & Depreeuw, 1998) and another about general psychology in collaboration with Eddy Van Avermaet and Paul Eelen (Lens, Van Avermaet, & Eelen, 1996), which have stimulated students, practitioners and scholars to think differently about motivation and psychology and were a source of inspiration for other Dutch text books on motivational dynamics (e.g., Vansteenkiste & Soenens, 2015).

Throughout his academic career, Willy sought to understand what moved people to act and to engage in their daily behaviors. To this end, he explored and contributed to expand many different theoretical frameworks in collaboration with his doctoral students and scholars worldwide. Although his primary interest was Time Perspective Theory, he contributed significantly to the refinement and expansion of Achievement Motivation, Achievement Goal perspectives, Expectancy x Value Theory, and most recently, Self-Determination Theory. His interest in human motivation was not limited to a single life domain, as he sought to study people’s motivational functioning in the fields of education, sports, health care, parenting, and (un)employment. Moreover, Willy showed a genuine interest in many areas of psychology beyond motivation, including experimental psychology, cross-cultural psychology and developmental psychology. His criterion to evaluate research was not its originating discipline, but its methodological rigor, incremental conceptual importance, and practical value in helping to understand real-life phenomena.

Indeed, despite his intense academic curiosity and his eagerness to conduct high quality research, he never lost sight of the application of high quality research. He was very well aware of the fact that theories and research needed to be eventually applied. Therefore, he scrutinized each of them, delved deep into its advantages and disadvantages and the circumstances under which one would be more applicable than other frameworks. He intellectually stimulated many psychologists, educators, parents, coaches, and leaders to reflect upon their own beliefs and their own motivating style and to consider their implications on their practice. When doing so, he urged practitioners to utilize theory to ground their instruction. Following Lewin (1952), he endorsed very much the adagio that “there was nothing more practical than a good theory.” Cartwright (1978) later added that “there was nothing more theoretical than an important practical problem.” Willy fully endorsed these beliefs, arguing that practitioners could very much challenge scholars in their theorizing, research, and writing (Lens, 1987; Lens, Matos & Herrera, submitted). Indeed, Willy strived for a synergistic collaboration with practitioners who, according to him, could enrich, sharpen, and refine his theory development and research, just as he would “push” collaborators to think about their work in complex and theoretically rich ways. He paved the way in Belgium and, truly, in many places over the world for motivational psychology to influence research in multiple domains and to influence practical applications of motivational theories in different settings. At the same time, he understood that real life is more complex than any theory can explain. As a consequence, he seldom considered a theory – any theory – as unmistakable or unquestionable.

His research collaborations went much beyond Belgium. In fact, he had connections with scholars on all continents. His collaboration represented several European countries spanning from Portugal to Poland and from the Netherlands and Norway to Turkey and Greece. But his collaborations went beyond Europe, also including Israel, Serbia, Russia, and Iran. In fact, he built deep and lasting connections with researchers in South-Africa, Peru, Japan, China, the US, Canada, Brazil, Chile, Costa Rica, and Australia, among others. The current special issue is also reflective of this worldwide network. Willy was internationally honored for his intellectual contributions as he was awarded honorary doctoral degrees from the Universidad Nacional Federico Villareal and the Pontificia Universidad Católica del Perú, both in Lima, Peru. Further, his personal contributions to research were acknowledged by two lifetime achievement awards. In 2010, the Special Interest Group on Motivation and Emotion of the European Association for Research on Learning and Instruction (EARLI) honored him with the “Oeuvre Award for Major Scientific Contributions to the field of Motivation and Emotion”. In 2014, he was awarded the “Inaugural Lifetime Achievement Award” at the International Conference on Time Perspective in Warsaw, Poland.

## Willy’s Personality: Contagious Enthusiasm, Honesty, and Lots of Generosity

Perhaps even more than through his academic work, it was the way Willy conducted research and developed his national and international network that impacted many of us. Willy’s enthusiasm and deep interest in psychology in general and motivation psychology in particular were contagious. It was no coincidence that Willy was particularly intrigued by the phenomenon of “intrinsic motivation” because he embodied it himself. His sincere curiosity in innovative research ideas and his excitement about findings or particular psychological phenomena had a stimulating impact on many scholars. Many people testify that they often felt energized and eager to learn more after having spoken with Willy. Even after his retirement, Willy continued to engage in interesting problems. He truly enjoyed mastering a new skill or resolving a problem, with his twinkling eyes signaling a combination of inherent satisfaction and pride when he achieved to do so. He took time to dig into the data, to study and grasp the peculiarities of different theories, and to fully understand how motivation works in “real life”.

Those who had the privilege to collaborate with him could attest that he welcomed scholars warmly to his office and took the time to fully understand and at the same time challenge their thinking. For many master students, doctoral students and collaborators, his office felt like a safe haven in the middle of the sometimes hectic academic world. This was because Willy listened with great care and attention, thereby raising our interest in the issues we discussed. Willy helped contextualize findings and ideas from a historical perspective, making sure that young scholars could ground their work in longstanding traditions.

Some of us have been Willy’s doctoral students and he challenged us by asking critical questions and by encouraging us not to take assumptions or methodological approaches for granted. He would frown when you told him that you adopted a design or a definition simply because they were commonly used in a research domain. Discussions with Willy could be very refreshing because he invited you to be critical of your perspective and to look beyond the customs in a research tradition. He liked to play the “devil’s advocate” and to defend a point of view opposite to your own, just for the sake of eliciting reflection. He did not necessarily want you to accept his point of view – Willy fully respected others’ viewpoint – but he invited you to deeply ponder, refine and possibly reconsider your perspective. He was a lively debater and he taught us to have thorough yet constructive discussions about research. While analyzing our data he encouraged us to immerse ourselves in it, advising us to first consider the basics before moving on to more sophisticated analyses. Some discussions with Willy were passionate and emotions sometimes ran high – other discussions were simply fun, as he brought in his own life, telling stories to make a point and many times just to make us laugh. We had such inspiring discussions and we miss these nowadays.

We also have known Willy as a committed, authentic and direct mentor and friend. He was sincere, straightforward in his comments and advice. His enthusiasm, but also his strong sense of justice, made him feel angry at times, particularly when he thought something was truthfully incorrect or unjust. When this happened we would all know about it. Sometimes, the issues were not of direct interest or importance to him. He could be very much concerned with the welfare of others or he could be fighting for a bigger goal he valued as a scientist or as a teacher.

Willy demonstrated the value of teaching and mentorship, nurturing dozens of scholars, preparing them to be part of an international community of researchers, and providing them with the support they needed throughout their academic career. As students graduated and grew into their own, Willy naturally shifted from being an advisor to a collaborator. Willy was as a generous, dedicated, and prosocial person. He was sincerely interested in other people and, due to his knowledge and skill, able to provide useful and well appreciated help. Many of us have experienced his continued availability and willingness to put as much effort as needed to support us, academically and personally. He has served on numerous doctoral committees, led dozens of seminars, and edited literally hundreds of manuscripts, even during the weekends. Even after his retirement, his faculty could count on his dedicated work. He took on these tasks selflessly and passionately. He made himself available to his students, his students’ students, colleagues, and to researchers from any part of the world who were asking for his guidance and assistance.

Willy earned enormous respect through his accessibility and authentic desire to support other people’s research rather than by referring to his academic status and prestige. Even long after his students obtained their PhD, he continued giving his support, whether that involved a quick chat, giving a piece of advice or an intense discussion of ideas. He wanted to contribute to the strengthening of the scientific work in several nations, especially those nations where the need for scientific support was the greatest. He made clear to us that one extra article would not make a difference, but that providing assistance and help to research communities in need of help could. These efforts were an example to us and made us consider the true value of our work as academics. We can only be grateful for his generosity and devoted mentorship. He was proud of our accomplishments, personal as well as professional, demonstrating a sincere interest and support for our partners, children, and families.

His openness to the world and his hospitality were equally an example to us. Not only he collaborated with scholars from all over the world, he also befriended them. He traveled across the world, but at the same time his own house was regularly filled with visitors coming from diverse continents. Visitors would find a warm welcoming home where they could learn about Flemish life and culture. Together with Hilde, his wife and life companion, he made his guests feel really at home. Willy and Hilde traveled together to see the world combining the hard work with sightseeing trips. They explored the world together, from the rainforest in South America, to traveling in a van across the US to going on a safari in South Africa. Together they enjoyed many opportunities for adventure.

This special issue in *Psychologica Belgica* is dedicated to Willy Lens, to honor an intellectual scholar, an inspiring mentor, and a great person. Despite our deep sadness for his premature death, we cannot but express our sincere gratitude to have known him, for being able to share our lives in many ways with him and for having gained so many positive memories thanks to him. Because we did not get the chance to say goodbye and to express our gratitude one last time, we wrote this tribute.

## Conclusion

Dr. Lens touched the hearts and influenced the lives of many people. He was not only considered a great professor and scholar, he was a true mentor, a friend, a most valued colleague, and even a father to many of us. His influence went far beyond the academic arena. Although his intellectual contributions will be reflected in the work of his students, colleagues, students’ students, and friends for many years to come, Willy’s generosity, modesty, humility, integrity, genuine interest in people, and sense of humor will be deeply missed by his family, colleagues, students, and all those who had the privilege to know and work with him. He devoted his life to the support of others’ development and growth. His caring attitude and mentorship and deep interest in motivation psychology will serve as a source of inspiration and as a role model for many of his students and fellows in the years to come. We can only express our deep gratitude to him for what he meant and still means in our lives. We will never forget him.

## References

[1] Cartwright D. (1978). Theory and practice. Journal of Social Issues.

[2] Cordeiro P. M., Paixão M. P., Lens W., Lacante M., Luyckx K. The Portuguese Validation of the Basic Psychological Need Satisfaction and Frustration Scale: Concurrent and longitudinal relations to well-being and ill-being. Psychologica Belgica.

[3] Lens W. (1987). Theoretical research should be useful and used. International Journal of Psychology.

[4] Lens W., Depreeuw E. (1998). Studiemotivatie en faalangst nader bekeken: tussen kunnen en moeten staat willen.

[5] Lens W., Matos L., Herrera D. (2016). Student motivation: Quantity and quality matter. Manuscript submitted for publication.

[6] Lens W., Van Avermaet E., Eelen P. (1996). Inleiding tot de psychologie.

[7] Lewin K. (1952). Field theory in social sciences: Selected theoretical papers by Kurt Lewin.

[8] Roman N. V., Davids E. L., Moyo A., Schilder L., Lacante M., Lens W. (2015). Parenting styles and psychological needs influences on adolescent life goals and aspirations in a South-African setting. Journal of Psychology in Africa.

[9] Vansteenkiste M., Soenens B. (2015). Vitamines voor groei: ontwikkeling voeden vanuit de Zelf-Determinatie Theorie.

